# The correlation between ethnicity and knowledge on nausea and vomiting in pregnancy among Arab and non- Arab women

**DOI:** 10.1186/s12884-026-09521-3

**Published:** 2026-07-27

**Authors:** Ibrahim Abu-Kishk, Rana Hajyahia, Yifat Wiener, Rana Cohen, Gideon Koren, Matitiahu Berkovitch, Miki Moskovich, Moshe Betser, Ilan Matok, Tomer Ziv-Baran, Dana Barchel

**Affiliations:** 1 Pediatric division, Faculty of Medical & Health Sciences, Shamir Medical Center (Assaf Harofeh), Tel Aviv University, Zerifin, Tel Aviv, Israel; 2 Division of Clinical Pharmacy, Institute for Drug Research, School of Pharmacy, Faculty of Medicine, Hebrew University of Jerusalem, Jerusalem, Israel; 3 Clinical Pharmacology and Toxicology Unit, Faculty of Medical & Health Sciences, Shamir Medical Center (Assaf Harofeh), Tel Aviv University, Zerifin, Tel Aviv, 7033001 Israel; 4 Maternal Fetal Unit, obstetrics and Gynecology Department, Faculty of Medical & Health Sciences, Shamir Medical Center (Assaf Harofeh), Tel Aviv University, Zerifin, Tel Aviv, Israel; 5 Kahn Maccabi Institute of Research and Innovation, Tel Aviv, Israel; 6 Division of Obstetrics and Gynecology, Faculty of Medical & Health Sciences, Shamir Medical Center (Assaf Harofeh), Tel Aviv University, Zerifin, Tel Aviv, Israel; 7 School of Public Health, Gray Faculty of Medical & Health Sciences, Tel Aviv University, Tel Aviv, Israel

**Keywords:** Hyperemesis gravidarum, Medical advice, Pregnancy, Nausea and vomiting of pregnancy

## Abstract

**Background:**

Nausea and vomiting in pregnancy (NVP) can substantially affect maternal well-being and quality of life. Cultural, social, and healthcare-system factors may influence healthcare-seeking behavior and management strategies. The primary objective of this study was to compare the proportion of Arab and non-Arab women who sought medical consultation for NVP. Secondary objectives were to assess differences in NVP duration and impact on quality of life, explore sources of advice and support, characterize recommendations for NVP management, and investigate reasons for not seeking medical consultation.

**Methods:**

This cross-sectional study was conducted in the maternity ward of Shamir (Assaf Harofeh) Medical Center between January and October 2018. Postpartum women who reported NVP during their most recent pregnancy were interviewed using a structured questionnaire available in Hebrew and Arabic. Group comparisons were performed using Chi-square, Fisher’s exact, t-tests, or Mann–Whitney U tests, as appropriate. Multivariable logistic regression was used to identify factors independently associated with seeking medical consultation.

**Results:**

A total of 190 women were included (126 non-Arab and 64 Arab). Overall, 92 non-Arab women (73.0%) sought medical consultation for NVP compared with 30 Arab women (46.9%) (*p* < 0.001). In the multivariable analysis, non-Arab ethnicity was independently associated with seeking medical consultation (OR = 2.64, 95% CI: 1.06–6.58; *p* = 0.036). Arab women reported greater NVP-related impairment in quality of life and daily functioning (*p* = 0.035) and were more likely to rely on family members for advice (45.3% vs. 28.6%, *p* = 0.022). Dietary modifications were recommended more frequently to Arab women (*p* = 0.023). No significant differences were observed between the groups regarding reasons for not seeking medical consultation.

**Conclusions:**

Significant differences were observed between Arab and non-Arab women in the perception and management of NVP. Arab women reported a greater impact of NVP on quality of life and were less likely to seek medical consultation, whereas non-Arab women more frequently sought professional care. Although ethnicity remained independently associated with healthcare-seeking behavior, the observed differences are likely influenced by a complex interplay of demographic, socioeconomic, cultural, behavioral, and healthcare-system-related factors. These findings highlight the importance of accessible, culturally sensitive, and patient-centered counseling to support equitable management of NVP.

## Introduction

Nausea and vomiting in pregnancy (NVP) are one of the most common pregnancy-related conditions, affecting approximately 50–80% of pregnant women worldwide [1]. Despite its high prevalence and substantial impact on maternal well-being, NVP has historically received limited attention from healthcare providers and researchers. In many settings, it is often perceived as a normal aspect of pregnancy that women are expected to manage independently [2].

The most severe form of the condition, hyperemesis gravidarum (HG), affects approximately 0.3–3.6% of pregnant women [3]. HG is characterized by persistent nausea and vomiting resulting in more than 5% loss of pre-pregnancy body weight, dehydration, ketonuria, and, in severe cases, electrolyte disturbances as well as thyroid or liver dysfunction [1, 4, 5]. If left untreated, HG may lead to serious maternal complications and adverse pregnancy outcomes [6, 7].

Beyond its physical manifestations, NVP has considerable psychosocial and economic consequences. It may significantly impair quality of life, affect family and social functioning, reduce work productivity, and contribute to psychological distress, including depression, anxiety, sleep disturbances, and emotional stress [8]. Furthermore, NVP imposes an economic burden through healthcare utilization and loss of workdays [9].

The etiology and clinical presentation of NVP are multifactorial, and epidemiological studies have reported inconsistent findings regarding factors associated with its occurrence, severity, and management. In particular, the influence of ethnicity and cultural background remains incompletely understood. While some studies have reported ethnic differences in the prevalence, severity, and management of NVP, others have found no significant associations [10–17].

Data regarding NVP among different ethnic groups in Israel are similarly inconclusive. One study reported lower rates of NVP and HG among Arab Bedouin women compared with non-Arab women, whereas another found no significant differences in prevalence between the two populations [18–20].

Arab citizens constitute approximately 20% of the Israeli population [21]. According to the Israeli Central Bureau of Statistics, Arab women, on average, marry at a younger age and are more likely to belong to lower socioeconomic strata than their non-Arab counterparts [22, 23]. These demographic and socioeconomic differences may influence health-related knowledge, perceptions of pregnancy symptoms, healthcare-seeking behavior, and approaches to the management of NVP.

Given the limited and conflicting evidence regarding ethnic differences in NVP and the lack of data addressing healthcare-seeking behavior and management strategies among Arab and non-Arab women in Israel, further investigation is warranted.

The primary objective of this study was to compare the proportion of Arab and non-Arab women who sought medical consultation for NVP. Secondary objectives were to:

- Assess differences in the severity and duration of NVP between the two populations.
- Examine the sources of consultation and support used by Arab and non-Arab women in coping with NVP.
- Characterize the recommendations provided for NVP management in each population.
- Explore reasons for not seeking medical consultation among women experiencing NVP.

## Materials and methods

This was a cross-sectional study based on personal interviews conducted in the maternity ward at Shamir (Assaf Harofeh) Medical Center. Participants were recruited consecutively from all eligible women who delivered at the hospital between January 1 and October 1, 2018. Shamir (Assaf Harofeh) Medical Center is a large tertiary medical center located in central Israel and serves a diverse population, including a substantial Arab population that routinely utilizes its obstetric services. Given the hospital’s catchment area and the broad utilization of its maternity services by both Arab and non-Arab women, the study population is considered to reasonably reflect the demographic composition of the region. Our objective was to investigate knowledge and cultural differences related to nausea and vomiting in pregnancy (NVP) while minimizing the potential confounding effect of unequal geographic access to healthcare services. Nevertheless, because participants were recruited using a convenience sampling approach at a single center, the possibility of selection bias cannot be entirely excluded.

The study included women in the early postpartum period who reported experiencing NVP during their most recent pregnancy. The rationale for selecting women in the early postpartum period who reported experiencing NVP was to enable assessment of NVP across the entire duration of pregnancy. This approach also allowed collection of end-of-pregnancy data regarding healthcare-seeking behavior, adherence to medical consultation, and the perceived effectiveness of consultations. The questionnaire was developed specifically for the study and was pilot tested prior to study initiation to assess clarity, comprehensibility, and feasibility. The questionnaire was available in both Hebrew and Arabic and underwent back-translation by an independent translator to ensure linguistic equivalence. To further support cultural appropriateness and accurate interpretation of the questions, all interviews were conducted by a bilingual interviewer whose native language was Arabic and who was also fluent in Hebrew.

Inclusion criteria were women who reported nausea and/or vomiting during their most recent pregnancy. Women with controlled chronic diseases (such as asthma, hypothyroidism, hypercoagulability, minor migraine and epilepsy) were included in the study. Exclusion criteria were pregnant women with multiple gestations, women who refused to participate, and those treated with chronic medications or who received fertility treatments during their last pregnancy and prior pregnancies.

**Fig. 1:**
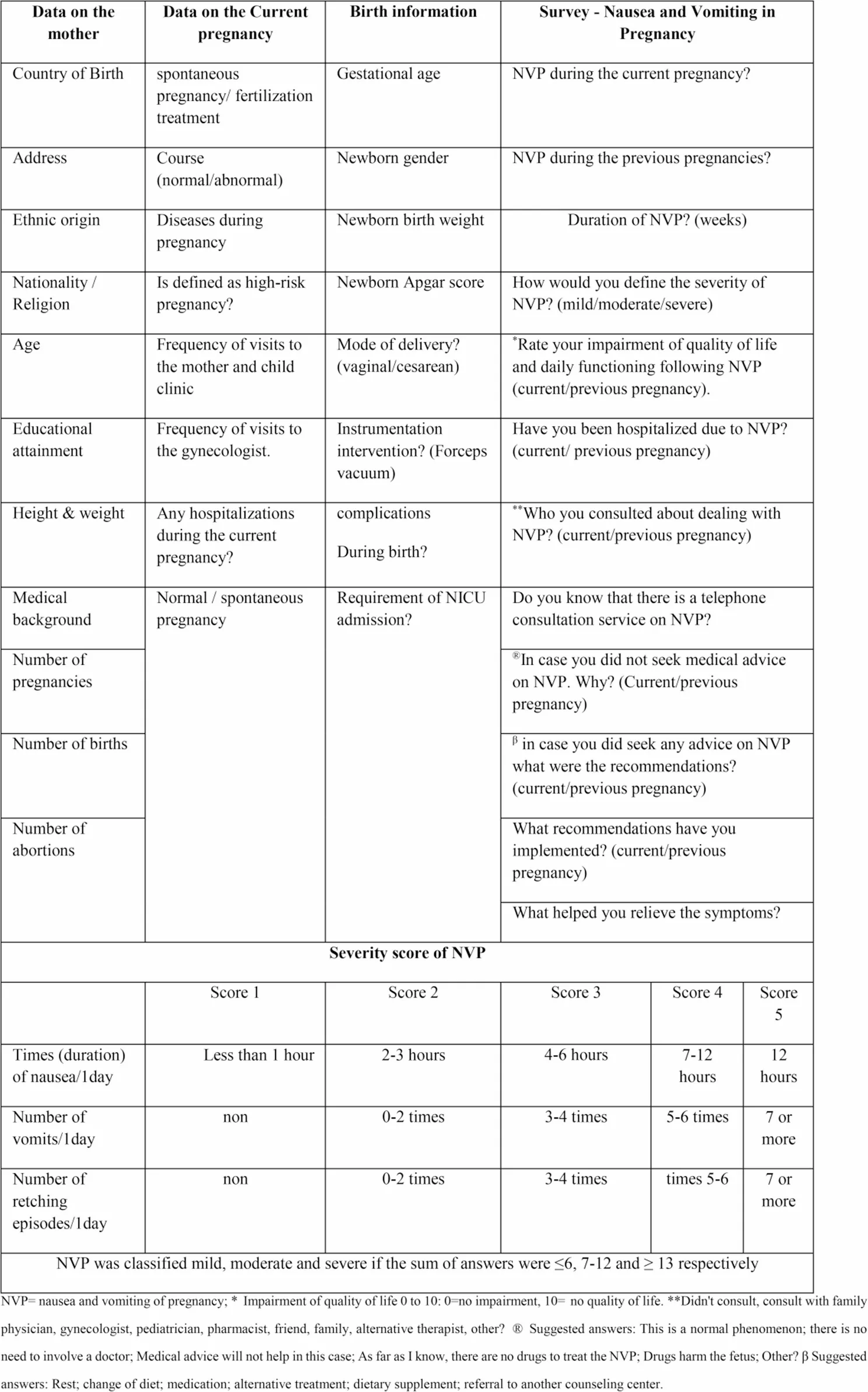
The study questionnaire

The questionnaire was designed to assess multiple aspects of NVP and its management. Comparisons between Arab and non-Arab women included NVP severity, quality of life, healthcare-seeking behavior, and recommendations received for symptom management. Additional items addressed current and previous pregnancies, reasons for not seeking medical consultation, treatment adherence, and perceived effectiveness of interventions (Fig. 1).

The following data were collected: maternal age, educational attainment (did not complete high school, completed secondary education, or higher education), pre-existing medical conditions, city of residence, and socioeconomic status based on the Israeli Central Bureau of Statistics socioeconomic index [22–24]. Obstetric variables included gravidity, parity, number of abortions, pregnancy-related complications, number of visits to obstetricians and family health clinics, high-risk pregnancy status, number of hospitalizations during the index and previous pregnancies, preterm birth (< 37 weeks), neonatal sex, birth weight, birth-weight percentile according to Dollberg growth curves for the Israeli population [25], birth complications, and Apgar scores.

A high-risk pregnancy was defined as any maternal or obstetric condition associated with an actual or potential risk to the health and well-being of the mother or fetus.

Quality of life was assessed using a Visual Analogue Scale (VAS) [26]. Participants rated the impact of NVP on overall quality of life on a scale ranging from 0 (no impairment) to 10 (maximum impairment). Additional items evaluated symptom-related factors, including poor appetite, symptom aggravation by specific foods or odors, fatigue, emotional distress, frustration, depressive symptoms, limitations in social activities, and difficulties performing daily tasks.

Severity of NVP was classified by the women as mild, moderate and severe using the Pregnancy-Unique Quantification of Emesis and Nausea (PUQE) score. The PUQE score is a validated clinical tool used to quantify the severity of NVP, including hyperemesis gravidarum. It is calculated based on three patient-reported parameters over a specified time frame (commonly 12–24 h): duration of nausea, number of vomiting episodes, and number of retching episodes. Each item is scored from 1 to 5, yielding a total score ranging from 3 (least severe) to 15 (most severe). Severity is categorized as mild (≤ 6), moderate (7–12), or severe (≥ 13) [27].

Advice-seeking behavior was defined as consultation with a physician, nurse, pharmacist, family member, or counseling service regarding NVP.

The study protocol and questionnaire were approved by the local Shamir Medical Center Ethics Committee (protocol number 0006-18-ASF).

### Sampling and analysis strategy

Because Arab citizens constitute approximately 20% of the Israeli population, it was initially assumed that the number of non-Arab women delivering at Shamir (Assaf Harofeh) Medical Center would be four times higher than the number of Arab women. In addition, it was estimated that 50% of non-Arab women experiencing NVP would seek medical consultation, compared with 20% of Arab women. Based on these assumptions, a sample of 29 Arab women and 116 non-Arab women (145 participants in total) was required to achieve 80% power at a significance level of 0.05.

To increase statistical power to 90% and reduce the significance level to 0.01, a sample of 51 Arab women and 204 non-Arab women (255 participants in total) was required. However, during recruitment, the observed ratio of non-Arab to Arab participants was approximately 2:1 rather than 4:1. Accordingly, the sample size calculation was revised, resulting in a required sample of 61 Arab women and 122 non-Arab women to achieve 90% power at a significance level of 0.01.

The primary outcome was seeking medical consultation for NVP. Independent variables included sociodemographic characteristics (age, ethnicity, educational attainment, and socioeconomic status), obstetric characteristics (gravidity and parity), and clinical characteristics related to NVP.

Categorical variables were summarized as frequencies and percentages. Normally distributed continuous variables were presented as mean ± standard deviation, whereas non-normally distributed variables were summarized as median and interquartile range. Univariate analyses were performed to identify factors associated with seeking medical consultation. Comparisons between Arab and non-Arab women were conducted using the Chi-square test or Fisher’s exact test for categorical variables, the independent-samples t-test for normally distributed continuous variables, and the Mann–Whitney U test for non-normally distributed continuous variables.

Multivariable logistic regression analysis was performed to identify factors independently associated with seeking medical consultation. Variables were selected based on clinical relevance, evidence from the literature, and statistical significance (*p* < 0.05) or borderline significance (*p* < 0.10) in univariate analyses [28]. Potential interactions between ethnicity and other variables included in the model were evaluated using an additional regression block with backward selection; no significant interactions were identified.

Data on educational attainment was missing for approximately 40% of participants. Missing values were addressed using multiple imputation with five iterations. Imputation was based on ethnicity, gravidity, parity, age, and socioeconomic status. Odds ratios (ORs) and corresponding confidence intervals were pooled across imputed datasets.

All statistical tests were two-sided, and *p* < 0.05 was considered statistically significant. Statistical analyses were performed using SPSS version 25 (IBM Corp., Armonk, NY, USA).

## Results

A total of 252 women were approached for participation in the study, of whom 34 declined, yielding a response rate of 86.5%. Among the non-respondents, 3 were Arab and 31 were non-Arab. No additional demographic or clinical information was available for these women.

**Fig. 2:**
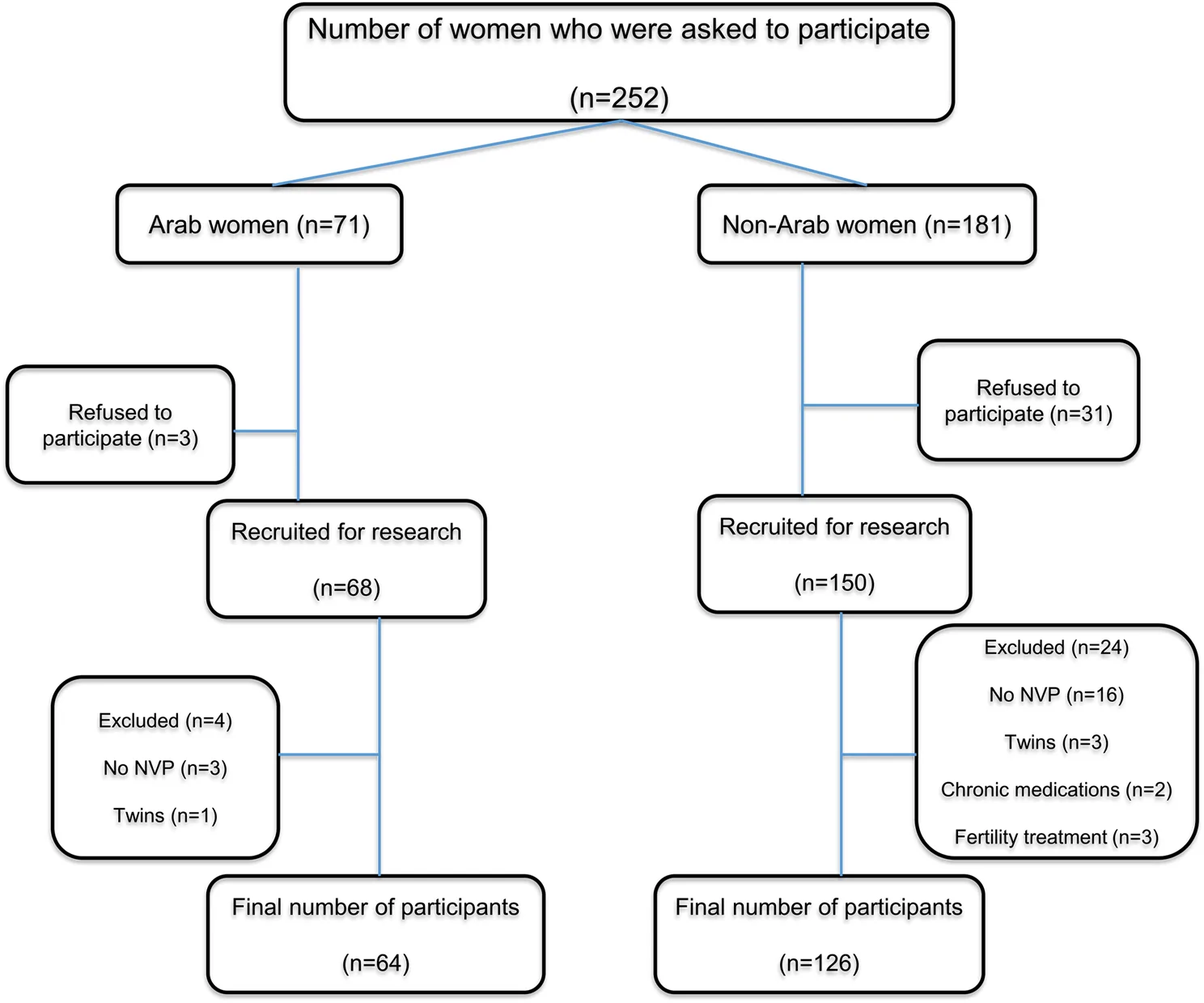
Population study flowchart

Of the 218 women who agreed to participate, 28 did not meet the eligibility criteria and were excluded from the analysis. The final study population therefore consisted of 190 women who reported NVP during their most recent pregnancy, including 64 Arab women and 126 non-Arab women (Fig. 2).

The median age of the study population was 28 years (Table 1). Non-Arab women were significantly older than Arab women (median age 30 vs. 25 years, respectively; *p* < 0.001). In addition, non-Arab women had a significantly higher socioeconomic status than Arab women (*p* < 0.001). The overall prevalence of high-risk pregnancies was 23.7% (*n* = 45), with no significant difference between the two groups.

**Table 1:** Participants characteristics

		Study group (*n* = 190)	Non-Arab (*n* = 126)	Arab (*n* = 64)	*p*-value
Maternal age, median (IQR), y		28 (24–33)	30 (26–34)	25 (21–31)	< 0.001
Underlying diseases, *n* (%)	Asthma	6 (3.2)	6 (4.8)	0 (0.0)	0.099
	Migraine	3 (1.6)	2 (1.6)	1 (1.6)	> 0.999
	Thyroid	4 (2.1)	4 (3.2)	0 (0.0)	0.302
	Hyper-coagulation	2 (1.1)	2 (1.6)	0 (0.0)	0.551
	Thalassemia	2 (1.1)	0 (0.0)	2 (3.1)	0.112
	Epilepsy	2 (1.1)	0 (0.0)	2 (3.1)	0.112
Number of Pregnancies, median (IQR)		2 (1–4)	2 (2–3)	2 (1–5)	0.689
Number of Births, median (IQR)		2 (1–3)	2 (1–3)	2 (1–4)	0.910
Number of Miscarriages, *n* (%)	0	136 (71.6)	93 (73.8)	43 (67.2)	0.322
	1	42 (22.1)	26 (20.6)	16 (25.0)	
	2	9 (4.7)	6 (4.8)	3 (4.7)	
	3	1 (0.5)	0 (0.0)	1 (1.6)	
	4	2 (1.1)	1 (0.8)	1 (1.6)	
Socioeconomic status, median (IQR)		4 (3–7)	7 (4–7)	3 (3–4)	< 0.001
Maternal education,* n* (%) ^*^	Did not complete high school	13 (11.3)	2 (2.5)	11 (30.6)	< 0.001
	High school education	39 (33.9)	24 (30.4)	15 (41.7)	
	Higher education	63 (54.8)	53 (67.1)	10 (27.8)	
Pregnancy course, *n* (%)	Normal	185 (97.4)	121 (96)	64 (100)	0.170
	Abnormal	5 (2.6)	5 (4.0)	0 (0.0)	
	Eclampsia	1 (0.5)	1 (0.8)	0 (0.0)	> 0.999
	Preeclampsia	6 (3.2)	3 (2.4)	3 (4.7)	0.406
	Diabetes	20 (10.5)	16 (12.7)	4 (6.3)	0.171
	Migraine	3 (1.6)	2 (2.9)	1 (0.8)	0.292
	DVT	1 (0.5)	1 (0.8)	0 (0)	> 0.999
	Cholestasis	1 (0.5)	1 (0.8)	0 (0)	> 0.999
Gestational weigh gain, median (IQR), kg		12 (8–16)	11 (8–16)	13 (9–17)	0.187
High risk pregnancy		45 (23.7)	33 (26.2)	12 (18.8)	0.254
Hospitalization during pregnancy, *n* (%)		54 (28.0)	28 (22.0)	26 (41.0)	0.008
community health clinic, *n* (%)		25 (13.4)	11 (8.9)	14 (22.6)	0.010
Average number of visits to a doctor monthly, *n* (%)	< 1	26 (14.0)	21 (16.9)	5 (8.1)	0.210
	1	104 (55.9)	67 (54.0)	37 (59.7)	
	1.5	2 (1.1)	1 (0.8)	1 (1.6)	
	2	39 (21.0)	27 (21.8)	12 (19.4)	
	2.5	1 (0.5)	0 (0)	1 (1.6)	
	3	11 (5.9)	8 (6.5)	3 (4.8)	
	4	3 (1.6)	0 (0)	3 (4.8)	
Number of visits to a gynecologist, median (IQR)		9 (6–10)	8 (6–10)	9 (8–10)	0.130

*IQR* interquartile range, *DVT* deep vein thrombosis, *y* years, *kg* kilograms, *n* number

* missing data in 47 non-Arab and 28 Arab women

### Primary outcome

Overall, 92 non-Arab women (73.0%) sought medical consultation for NVP, compared with 30 Arab women (46.9%) (*p* < 0.001) (Table 2).

**Table 2:** medical consultation regarding NVP in the non-Arab and Arab population

Variable	Study group (*n* = 190)	Non- Arab Women (*n* = 126)	Arab women (*n* = 64)	*P*-value
Medical consultation (Doctor, Pharmacist, Nurse, Telephone-counseling), *n* (%)	122 (64.2)	92 (73)	30 (46.9)	< 0.001

	*N* = 111	*N* = 76	*N* = 35	
Medical consultation in previous pregnancies (Doctor, Pharmacist, Nurse, Telephone-counseling), *n* (%)	72 (64.9)	54 (71.1)	18 (51.4)	0.004

Hospitalization during pregnancy was significantly more common among Arab women, with 26 women (40.6%) requiring hospital admission compared with 28 women (22.2%) in the non-Arab group (*p* = 0.008). Arab women were also more likely to seek care at community health clinics during pregnancy, with 14 women (22.6%) reporting clinic visits compared with 11 women (8.9%) among non-Arab women (*p* = 0.01) (Table 1).

### Secondary outcomes

#### Differences in the severity and duration of NVP between Arab and non-Arab women

Table 3 summarizes the characteristics of NVP, including symptom duration and perceived severity. In most participants, symptoms resolved spontaneously during pregnancy, with a median gestational age at symptom resolution of 16 weeks. No significant differences were observed between Arab and non-Arab women regarding the timing of symptom resolution.

**Table 3:** Duration and severity of NVP in the study group

Variable		Study Group	Non-Arabs	Arabs	*P*-value
		(*n* = 190)	(*n* = 126)	(*n* = 64)	
Gestational age (weeks) at cessation of symptoms, median (IQR), week		16 (12–25)	16 (12–25)	16 (12–37)	0.689
Symptoms continued until delivery, *n* (%)		37 (19.5)	20 (15.9)	17 (26.6)	0.079
Severity of NVP, *n* (%)	Mild	41 (21.6)	29 (23)	12 (18.8)	0.014
	Moderate	96 (50.5)	71 (56.3)	25 (39.1)	
	Severe	53 (27.9)	26 (20.6)	27 (42.2)	
Impaired daily functioning and quality of life score, median (IQR)		3 (5–8)	5 (2–7)	6 (3–10)	0.035
Maximum Impairment of daily functioning and quality of life, *n* (%)		32 (16.8)	16 (12.7)	16 (25.0)	0.032
No Impairment of daily functioning and quality of life, *n* (%)		33 (17.4)	26 (20.6)	7 (10.9)	0.095
Impaired daily functioning and quality of life score in previous pregnancies, median (IQR)		3 (5–8)	5 (3–7)	7 (5–10)	0.017
Hospitalization due to NVP, *n* (%)		4 (2.1)	3 (2.4)	1 (1.6)	> 0.999

		(*n* = 131)	(*n* = 92)	(*n* = 39)	
Hospitalization due to NVP in previous pregnancies, *n* (%)		2 (1.6)	2 (2.3)	0 (0)	> 0.999
NVP prevalence in previous pregnancies, *n* (%)		112 (85.5)	77 (83.7)	35 (89.7)	0.369

*NVP* nausea and vomiting of pregnancy, *IQR* interquartile range, *n* number

Overall, 37 women (19.5%) reported persistence of symptoms until delivery. Although symptom persistence was more frequently reported among Arab women than among non-Arab women (26.6% vs. 15.9%), the difference did not reach statistical significance (*p* = 0.079).

Assessment of NVP-related impairment in quality of life and daily functioning using a 10-point scale demonstrated higher median scores among Arab women compared with non-Arab women (6 vs. 5, respectively; *p* = 0.035). Severe impairment, defined as the maximum score on the scale, was reported by 25.0% of Arab women and 12.7% of non-Arab women (*p* = 0.032).

#### Sources of consultation and Support for NVP

Overall, 122 women (64.2%) reported seeking advice or consultation regarding NVP during their most recent pregnancy. Consultation with physicians was significantly more common among non-Arab women than among Arab women (69.0% vs. 40.6%, respectively; *p* < 0.001). In contrast, Arab women were more likely to seek advice from family members (45.3% vs. 28.6%; *p* = 0.022). Consultation with friends was reported more frequently by non-Arab women (21.4% vs. 7.8%; *p* = 0.018).

#### Recommendations for NVP management

**Fig. 3:**
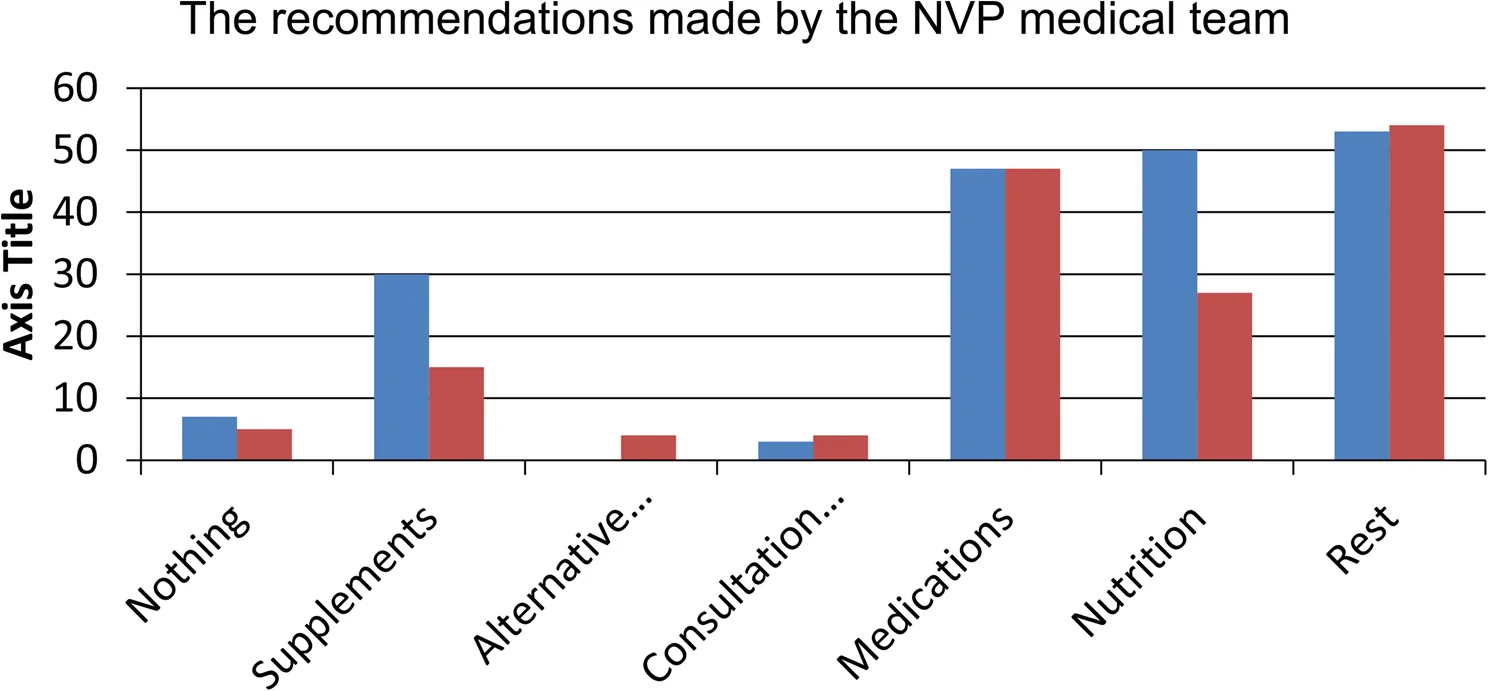
Distribution of the recommendations made by the NVP medical team

Recommendations provided by healthcare professionals included rest, dietary modifications, pharmacological treatment, referral to a dedicated NVP counseling service, complementary or alternative therapies, and nutritional supplements (Fig. 3).

Apart from dietary modifications, which were recommended more frequently to Arab women than to non-Arab women (*n* = 50 vs. *n* = 25, respectively; *p* = 0.023), no significant differences were observed between the groups with respect to other recommendations, including rest, pharmacological treatment, referral to an NVP counseling service, complementary therapies, or nutritional supplements.

Regarding implementation of recommendations, a significant difference between-group was observed only for dietary modifications. Arab women were more likely than non-Arab women to report adopting dietary changes as part of NVP management (51.7% vs. 23.3%, respectively; *p* = 0.004). Approximately 40% of women who sought medical consultation reported using pharmacological treatment for NVP, with no significant difference between Arab and non-Arab women (37.9% vs. 40.7%, respectively; *p* = 0.79).

There was a trend toward a higher proportion of Arab women reporting symptom relief following dietary modifications compared with non-Arab women (43.3% vs. 25.3%; *p* = 0.061), although this difference did not reach statistical significance. Relief following pharmacological treatment was reported by 31.4% of participants, the majority of whom were non-Arab women (32 of 38 participants). Only three women reported benefit from complementary therapies. Nutritional supplements were perceived as beneficial by 13.2% of non-Arab women and 3.3% of Arab women; however, this difference was not statistically significant (*p* = 0.182).

Overall, 21 women (17.4%) reported no relief from NVP symptoms despite the interventions used. This was reported more frequently by Arab women than by non-Arab women (30.0% vs. 13.2%, respectively; *p* = 0.035).

In univariate analyses, non-Arab ethnicity (*p* < 0.001), higher socioeconomic status (*p* = 0.02), and longer duration of NVP symptoms (*p* = 0.006) were associated with seeking medical consultation (Table 4).

**Table 4:** Univariate analysis of factors affecting medical consultation

Variable		Arab women	Non-Arab women	*p*-value
Medical consultation, *n* (%)		30 (46.8%)	92 (71.4%)	**< 0.000**
Maternal age, median (IQR), y		28 (23–34)	29 (25–33)	0.314
Underlying diseases, *n* (%)	Asthma	1 (1.5%)	5 (4.1)	0.423
	Migraine	2 (2.9%)	1 (0.8)	0.292
	Thyroid	2 (2.9%)	2 (1.6)	0.618
	Hyper-coagulation	1 (1.5%)	1 (0.8)	> 0.999
	Thalassemia	0 (0.0%)	1 (1.5)	> 0.999
	Epilepsy	1 (1.5%)	1 (0.8)	> 0.999
Number of Pregnancies, median (IQR)		2 (2–5)	2 (1–3)	0.082
Socioeconomic status, median (IQR)		4 (3–7)	5.5 (4–7)	0.020
Pregnancy course, *n* (%)	Normal	65 (95.6)	120 (98.4)	0.351
	Abnormal	3 (4.4)	2 (1.6)	
Diseases during pregnancy, *n* (%)	Ecalmpsia	1 (1.5)	0 (0.0)	0.358
	Preeclampsia	1 (1.5)	5 (4.1)	0.423
	Diabetes	6 (8.8)	14 (11.5)	0.568
	Migraine	2 (2.9)	1 (0.8)	0.292
	DVT	1 (1.5)	0 (0)	0.358
	Cholestasis	0 (0)	1 (0.8)	> 0.999
Gestational weigh gain, median (IQR), kg		12 (7–16)	11 (9–16)	0.707
High Risk Pregnancy		11 (16.2)	34 (27.9)	0.069
Hospitalization During Pregnancy, *n* (%)		18 (26.5)	36 (30)	0.656
Gestational age at cessation of symptoms, median (IQR), wk.		14 (12–17)	17 (12–35)	0.006
Severity of NVP, *n* (%)	Mild	19 (27.9)	22 (18.0)	0.103
	Moderate	32 (47.1)	64 (52.5)	
	Severe	17 (25.0)	36 (29.5)	
Impaired daily functioning and quality of life score, median (IQR)		5 (2–7)	5 (3–8)	0.301
Hospitalization due to NVP, *n* (%)		1 (1.6)	3 (2.4)	0.299
Hospitalization due to NVP in previous pregnancies, *n* (%)		0 (0)	2 (2.5)	0.522
Years of Education, median (IQR), y, *		12.5 (12–16)	15 (12–16)	0.329

*IQR* Interquartile range, *wk* weeks, *DVT* deep vein thrombosis, *NVP* nausea and vomiting of pregnancy, *y* years, *n* number

* missing data in 47 non-Arab and 28 Arab women

In the multivariable logistic regression analysis, non-Arab ethnicity was independently associated with seeking medical consultation for NVP. Non-Arab women had approximately 2.6-fold higher odds of seeking medical consultation compared with Arab women (OR = 2.64, 95% CI: 1.06–6.58; *p* = 0.036).

Parity was inversely associated with seeking medical consultation, with each additional birth associated with a 25% reduction in the odds of seeking consultation (OR = 0.75, 95% CI: 0.61–0.93; *p* = 0.010).

Longer duration of NVP symptoms was also independently associated with seeking medical consultation (OR = 1.04, 95% CI: 1.01–1.08; *p* = 0.021), indicating that women with more persistent symptoms were more likely to seek professional care (Table 5).

**Table 5:** Multivariate analysis of factors affecting medical advice

	OR	95% CI for OR		*P*-value^®^
		Lower	Upper	
Non-Arabs	2.645	1.063	6.579	0.036
Age (years)	1.067	0.992	1.148	0.083
Number of pregnancies^*^	0.751	0.605	0.933	0.010
Gestational age at cessation of symptoms (weeks)^α^	1.043	1.006	1.081	0.021
High risk pregnancy	1.562	0.677	3.603	0.296
Severity of NVP- Mild	1			0.596
Severity of NVP- Moderate	1.49	0.639	3.472	
Severity of NVP- Severe	1.602	0.571	4.492	
Socioeconomic status	1.005	0.779	1.298	0.968
Years of education	0.985	0.829	1.169	0.885

^®^ logistic regression; *The higher the number, the lower the rate; α- The longer the symptoms the higher the rate

Overall, 68 women (35.8%) reported not seeking medical consultation for NVP during their most recent pregnancy. This proportion was significantly higher among Arab women than among non-Arab women (53.1% vs. 26.9%, respectively) (Table 2).

The most frequently reported reason for not seeking medical consultation was the perception that NVP is a normal and expected part of pregnancy. No significant differences were observed between Arab and non-Arab women in the distribution of reasons for not seeking medical consultation (Table 6).

**Table 6:** reasons for not approaching medical consultation to treat NVP

variable		Study group (*n* = 67)	Non-Arab (*n* = 33)	Arab (*n* = 34)	*P*-value
Reasons why women did not seek medical counseling, *n* (%)	Normal phenomenon	58 (86.6)	27 (81.8)	31 (91.2)	0.305
	Will not help	11 (16.4)	6 (18.2)	5 (14.7)	0.701
	No medication for NVP	5 (7.5)	4 (12.1)	1 (2.9)	0.197
	Medications are unsafe for the fetus	5 (7.5)	3 (9.1)	2 (5.9)	0.672

## Discussion

In this study, we identified significant differences between Arab and non-Arab women in the experience, perception, and management of nausea and vomiting in pregnancy (NVP). Non-Arab women were generally older and belonged to higher socioeconomic strata, and they were significantly more likely to seek medical consultation for NVP. In contrast, Arab women more frequently relied on family members for support. In multivariable analysis, non-Arab ethnicity and longer symptom duration were independently associated with higher rates of medical consultation, whereas increasing parity was associated with lower rates of medical consultation.

The demographic differences observed between the groups, including younger age and lower educational attainment among Arab women, are consistent with previously reported demographic and socioeconomic patterns within the Israeli population [21]. Our findings also align with previous studies demonstrating differences in healthcare utilization between Arab and non-Arab populations in Israel, including lower use of specialist consultations, prenatal diagnostic services, and genetic testing among Arab women [29–31]. However, these observations should be interpreted with caution, as healthcare-seeking behaviors are influenced by multiple interrelated factors.

Although differences in education and socioeconomic status were evident in the univariate analysis, these variables did not remain independently associated with healthcare consultation in the multivariable models. Similar findings have been reported previously, suggesting that disparities in healthcare utilization cannot be fully explained by socioeconomic factors alone [30, 32]. The observed patterns may reflect a complex interaction between cultural beliefs, health literacy, social support networks, accessibility of healthcare services, language considerations, trust in healthcare providers, and other structural determinants of health. Therefore, ethnic differences in healthcare utilization should not be attributed solely to cultural preferences but rather considered within the broader social and healthcare context. Regarding the clinical course of NVP, the median gestational age at symptom resolution was 16 weeks, which is consistent with previous reports [5, 33]. Approximately one-fifth of participants reported symptoms that persisted until delivery. Arab women reported a greater impact of NVP on quality of life and daily functioning compared with non-Arab women. Previous studies have demonstrated ethnic variation in the prevalence and severity of NVP, although the available evidence remains inconsistent [17, 19]. The differences observed in our cohort may be influenced by a range of factors, including socioeconomic conditions, dietary patterns, health perceptions, symptom reporting, and cultural attitudes toward pregnancy-related symptoms. Further studies are required to better understand the mechanisms underlying these differences.

Hospitalization rates for hyperemesis gravidarum (HG) did not differ significantly between the groups, and the overall prevalence was comparable to rates reported in both Israeli and international populations [4, 19]. Arab women more frequently reported benefit from dietary modifications, whereas non-Arab women more commonly reported improvement following the use of medications and nutritional supplements. These findings may reflect differences in treatment preferences, access to healthcare resources, counseling practices, or perceptions regarding treatment effectiveness. However, the cross-sectional design of the study precludes definitive conclusions regarding the reasons for these differences.

Although nearly all women were followed by obstetricians during pregnancy, Arab women more frequently reported receiving care at family health clinics, which are primarily nurse-led. This pattern may reflect differences in healthcare accessibility, service availability, referral pathways, familiarity with community-based healthcare resources, or patient preferences. These findings highlight the important role of community healthcare providers in supporting pregnant women and suggest that strengthening community-based services may improve patient engagement and health outcomes. Notably, nearly one-third of Arab women reported no symptom relief despite adherence to recommendations, compared with only 13% of non-Arab women. This discrepancy may be related to differences in treatment effectiveness, communication with healthcare providers, expectations regarding symptom improvement, or other unmeasured factors, and warrants further investigation.

Despite reporting more severe symptoms and greater impairment in quality of life, Arab women were less likely to seek medical consultation and more likely to regard NVP as a normal part of pregnancy. While cultural perceptions may contribute to this pattern, alternative explanations should also be considered. Variations in healthcare utilization may reflect differences in healthcare accessibility, transportation availability, health literacy, competing family responsibilities, previous experiences with the healthcare system, or other structural and socioeconomic factors that were not fully captured in the present study. Consequently, the lower consultation rates observed among Arab women should not be interpreted solely as a reflection of cultural attitudes but rather as the result of multiple interacting influences.

Fear of medication-related teratogenicity, which has been identified in previous studies as an important barrier to treatment [34–36], was reported by only a small number of participants in our cohort. This finding may reflect increasing awareness regarding the safety of recommended treatments for NVP or may be related to local counseling practices.

Finally, Arab women were more frequently hospitalized during pregnancy. This observation is consistent with previous reports that have identified associations between patterns of healthcare utilization and hospitalization during pregnancy [37]. However, given the observational nature of the study, causality cannot be inferred. Differences in hospitalization rates may also be influenced by disease severity, healthcare access, referral practices, socioeconomic circumstances, and other unmeasured confounding factors.

Overall, our findings suggest that significant differences exist between Arab and non-Arab women in the perception and management of NVP. These differences appear to reflect a combination of demographic, social, healthcare-system, and potentially cultural factors. Future studies should further explore the mechanisms underlying these disparities and evaluate interventions aimed at improving equitable access to information, counseling, and evidence-based treatment for NVP across diverse populations.

### Strengths and limitations

This study is the first in Israel to systematically examine the influence of ethnicity on NVP-related health-seeking behavior. Its strengths include the use of a structured questionnaire that was pilot tested, translated and back translated, and administered in the participants’ native language by a bilingual interviewer, thereby supporting linguistic and cultural appropriateness. In addition, the study evaluated both symptom burden and healthcare utilization patterns.

However, several limitations should be acknowledged. First, the study relied on self-reported data collected after participants had experienced nausea and vomiting during pregnancy. Therefore, the findings may be subject to recall bias, particularly regarding symptom severity, duration, treatment effectiveness, and healthcare-seeking behaviors.

Second, this was a single-center study conducted at a tertiary medical center in Israel. Consequently, local demographic characteristics, healthcare practices, and referral patterns may have influenced the findings. As a result, the observed associations may not fully reflect experiences in other regions or healthcare settings.

Third, although the sample size was sufficient to identify several significant associations, it may have limited the statistical power to detect smaller differences between groups and to evaluate potential interactions among demographic, socioeconomic, and clinical variables.

In addition, despite adjustment for several covariates in the multivariable analyses, residual confounding cannot be excluded. Factors such as health literacy, healthcare accessibility, transportation availability, social support, language barriers, and other social determinants of health were not directly measured and may have influenced healthcare utilization patterns.

Finally, the study population consisted of women attending a specific healthcare setting, which may limit the generalizability of the findings. Therefore, caution should be exercised when extrapolating these results to the broader population of pregnant women in Israel or to populations in other countries with different cultural, socioeconomic, and healthcare-system characteristics.

### Clinical implications

Our findings highlight the importance of accessible, culturally sensitive, and patient-centered counseling strategies for women experiencing NVP. Recognition of cultural, social, and healthcare-system-related determinants is essential to improve access to information, facilitate timely consultation, and optimize symptom management. Tailored health education, enhanced community-based services, and proactive involvement of healthcare providers, particularly nurses in family health clinics, may improve access to care and maternal well-being.

## Conclusion

This study identified significant differences between Arab and non-Arab women in the experience, perception, and management of nausea and vomiting in pregnancy (NVP). Arab women reported a greater impact of NVP on quality of life and daily functioning and were less likely to seek medical consultation, while more frequently relying on family support. In contrast, non-Arab women more often consulted healthcare professionals and reported greater benefit from pharmacological treatments and nutritional supplements, whereas Arab women more commonly adopted dietary modifications.

Although non-Arab ethnicity remained independently associated with healthcare-seeking behavior in the multivariable analysis, the observed differences should be interpreted with caution. They are likely influenced by a complex interplay of demographic, socioeconomic, cultural, behavioral, and healthcare-system-related factors. Furthermore, residual confounding from unmeasured variables, including health literacy, healthcare accessibility, and social determinants of health, cannot be excluded.

These findings highlight the importance of providing accessible, culturally sensitive, and patient-centered counseling regarding NVP, while ensuring equitable access to healthcare services. Further research is needed to better understand the factors underlying differences in healthcare utilization and to identify interventions that improve maternal care and quality of life across diverse populations.

## Data Availability

No datasets were generated or analysed during the current study.
